# Deletion of suppressor of cytokine signaling 3 (SOCS3) in muscle stem cells does not alter muscle regeneration in mice after injury

**DOI:** 10.1371/journal.pone.0212880

**Published:** 2019-02-27

**Authors:** Kristy Swiderski, Marissa K. Caldow, Timur Naim, Jennifer Trieu, Annabel Chee, René Koopman, Gordon S. Lynch

**Affiliations:** Centre for Muscle Research, Department of Physiology, The University of Melbourne, Victoria, Australia; University of Tennessee Health Science Center College of Graduate Health Sciences, UNITED STATES

## Abstract

Muscles of older animals are more susceptible to injury and regenerate poorly, in part due to a persistent inflammatory response. The janus kinase (Jak)/signal transducer and activator of transcription (Stat) pathway mediates inflammatory signaling and is tightly regulated by the suppressor of cytokine signaling (SOCS) proteins, especially SOCS3. SOCS3 expression is altered in the muscle of aged animals and may contribute to the persistent inflammation and impaired regeneration. To test this hypothesis, we performed myotoxic injuries on mice with a tamoxifen-inducible deletion of SOCS3 specifically within the muscle stem cell compartment. Muscle stem cell-specific SOCS3 deletion reduced muscle mass at 14 days post-injury (-14%, *P* < 0.01), altered the myogenic transcriptional program, and reduced myogenic fusion based on the number of centrally-located nuclei per muscle fiber. Despite the delay in myogenesis, muscles with a muscle stem cell-specific deletion of SOCS3 were still able to regenerate after a single bout or multiple bouts of myotoxic injury. A reduction in SOCS3 expression in muscle stem cells is unlikely to be responsible for the incomplete muscle repair in aged animals.

## Introduction

Successful skeletal muscle repair is essential for the maintenance of muscle integrity to maintain quality of life. When injured, damaged muscle fibers release factors that promote recruitment of inflammatory cells and the activation and proliferation of muscle stem cells. Activated muscle stem cells proliferate, migrate, and fuse to repair damaged muscle fibers in a process highly dependent on a properly regulated inflammatory response [1]. In drosophila, the family member Tinman was discovered to be a major regulator of cell fate and muscle development via the Janus kinase (Jak)/Signal transducers and activators of transcription (Stat) Jak/Stat signaling pathway [2]. Since then, Jak/Stat signaling has been shown to regulate muscle stem cell activity, as mice with a muscle stem cell specific deletion of STAT3 demonstrate impaired myogenesis resulting from altered myogenic fusion [3].

One key family of negative regulators of Jak/Stat signaling are the suppressor of cytokine signalling (SOCS) proteins. Of the eight members of the SOCS protein family [cytokine-inducible SH2-containing protein (CISH) and SOCS1-7], SOCS3 is the best characterised in skeletal muscle [4–9]. Gene expression analyses in mice showed significantly higher *Socs3* gene expression in freshly isolated ‘quiescent’ versus *in vitro* activated muscle stem cells, suggesting a potential role for SOCS3 in maintaining quiescence [10, 11]. Additionally, in the C2C12 myogenic cell line, SOCS3 promotes myogenic differentiation by modulating the leukemia inhibitory factor (LIF) and insulin-like growth factor (IGF-1) signaling pathways [5, 8]. Regulation of Jak/Stat signaling by SOCS3 is therefore likely to be important for successful progression through myogenesis.

Muscles of old animals are more susceptible to injury and regenerate poorly resulting in incomplete functional recovery, a process linked to a persistent inflammatory response [12, 13]. As the Jak/Stat signaling pathway is a major mediator of the inflammatory response in skeletal muscle, dysregulated Jak/Stat signaling results in persistent inflammation [14–18]. Increased STAT3 signaling in old skeletal muscle has been commonly reported [6, 19, 20], suggesting that the negative regulation of Jak/Stat signaling by SOCS3 is impaired. Consistent with these observations, Jak/Stat signaling is increased in the muscle stem cell population of aged (18 month old) relative to young (3 week old) mice [21], indicating dysregulation of Jak/Stat signalling.

Thus, SOCS3 may play a regulatory role during myogenesis and altered levels of SOCS3 in old muscles might impair the regenerative response. As multiple cell types within regenerating skeletal muscles express SOCS3, including the muscle fibers, inflammatory cells and the muscle stem cells, the relative contribution of SOCS3 within these cell types to altered muscle inflammation and regeneration remains to be determined. We previously reported that specific deletion of SOCS3 in mature skeletal muscle fibers enhances the inflammatory response after myotoxic injury but does not impair regeneration [9]. Using mice lacking SOCS3 specifically within Pax7-expressing muscle stem cells, we now test the hypothesis that deletion of SOCS3 within the muscle stem cell population delays muscle regeneration after myotoxic injury.

## Materials and methods

### Animals

B6.Cg-*Pax7*^*tm1(cre/ERT2)Gaka*^/J (Pax7-CreER) mice were obtained from The Jackson Laboratory (Bar Harbor, ME, USA) and mated to SOCS3^fl/fl^ mice (obtained originally from Prof. Gregory Steinberg, St Vincent’s Institute for Medical Research, Melbourne, Australia). Breeding generated animals homozygous for Cre-recombinase expression (SOCS3^fl/fl^ Pax7-CreER^+^ mice) and Cre-negative controls (SOCS3^fl/fl^ Pax7-CreER^-^ mice). All mice were bred and maintained in the Biological Research Facility (BRF) at The University of Melbourne, Australia. To induce Cre-recombinase expression, ten-week-old male and female control (SOCS3^fl/fl^ Pax7-CreER^-^) and SOCS3 MscKO (SOCS3^fl/fl^ Pax7-CreER^+^) mice received daily intraperitoneal (*i*.*p)* administration of tamoxifen (Sigma Aldrich, St. Louis, MO, USA; 200 μL of 10 mg/mL tamoxifen in corn oil) for 5 d and experiments commenced 14 d after the first tamoxifen injection. All experimental protocols were approved by the Animal Ethics Committee of The University of Melbourne, Australia and conducted in accordance with the Australian code of practice for the care and use of animals for scientific purposes as stipulated by the National Health and Medical Research Council (Australia).

### Myotoxic injury

Following tamoxifen administration, twelve-week-old male and female control and SOCS3 MscKO were anesthetized with 100 mg/kg ketamine (*i*.*p;* Ceva Animal Health Pty. Ltd., Glenorlie, NSW, Australia) and 10 mg/kg xylazine (ilium xylazil-20; Troy Laboratories, Smithfield, NSW, Australia) and received an injection of notexin into the right tibialis anterior (TA) muscle. Mice were killed at 1, 2, 3, 7, or 14 d after notexin injury, as described previously [9]. All muscles were subsequently stored at -80°C.

### Confirmation of SOCS3 deletion in muscle stem cells

To confirm SOCS3 deletion within the muscle stem cell compartment, the right TA muscle of tamoxifen-treated control and SOCS3 MscKO mice was injected with 40 μl notexin (10 μg/ml saline; Latoxan, Valence, France) to induce muscle fiber degeneration. At 28 d post-notexin injury, mice received an *i*.*p*. injection of either saline (n = 2) or lipopolysaccharide (LPS; n = 2) to induce *Socs3* gene expression. At 4 h after injection, mice were killed by cervical dislocation and the right TA muscles dissected and snap frozen. Total RNA was extracted from each right TA muscle (n = 6/genotype/timepoint) using an RNeasy Fibrous Tissue Mini Kit (Qiagen, Venlo, Limburg, Netherlands) as per manufacturer’s instructions, converted to cDNA and analyzed by qPCR for *Socs3* gene expression.

### Assessment of skeletal muscle contractile properties

At 7 d post-notexin injury, both injured and uninjured mice were anesthetized with sodium pentobarbitone (Nembutal; 60 mg/kg; Sigma-Aldrich) *via i*.*p*. injection and contractile properties were assessed as described previously [9, 22].

### Histology

Serial sections (5 μm) were cut transversely through the TA muscle using a refrigerated (−20°C) cryostat (CTI Cryostat; IEC, Needham Heights, MA). Sections were stained with hematoxylin and eosin (H&E) and digital images of stained sections obtained using an upright microscope with camera (Axio Imager day 1, Carl Zeiss, Wrek, Göttingen, Germany), controlled by AxioVision AC software (AxioVision AC Rel. 4.8, Carl Zeiss Imaging Solutions, Wrek, Göttingen, Germany) as described previously [9].

### Immunofluorescence

Sections of TA muscle (5 μm) were fixed for 10 min in methanol at -20°C, air-dried and incubated with Alexa488-conjugated Anti-F4/80 (ab204266; 1:100; Abcam, Cambridge, UK) and Alexa647-conjugated Anti-CD68 (ab201845; 1:200; Abcam, Cambridge, UK) antibodies for 1 h at room temperature in a humidified chamber. Slides were rinsed for 5 min in PBS containing 0.05% Tween20 (PBStw) and 2 × 5 min in PBS and then incubated for 30 min with 4’,6-diamindino-2-phenylindole (DAPI, 5 μg/mL PBS) to visualize nuclei. For Pax7 immunostaining, sections were fixed in 4% PFA for 10 min and rehydrated in 0.1% PBStw for 2 × 5 min. Heat activated antigen retrieval was performed in citrate buffer (pH 6.0) in the high-pressure cooker for 10 min, slides were cooled to RT and washed in 0.1% PBStw for 2 × 5 min. Sections were blocked in 10% Affinipure FAB goat anti mouse IgG/3% bovine serum albumin (BSA; in 0.1% PBStw) for 45 min at RT, then washed with PBStw for 2 × 5 min and incubated in anti-PAX7-s (pax7, RRID:AB_528428, developed by A. Kawakami from the Tokyo Institute of Technology and obtained from the Developmental studies hybridoma bank, Iowa City, IO; 1:10) and anti-Laminin (#L9393; Sigma-Aldrich, St. Louis, MO, USA; 1:50) in 3% BSA (in 0.1% PBStw) overnight at 4°C. Slides were rinsed with 0.1% PBStw for 3 × 10 min and incubated with secondary antibodies (Goat anti-mouse IgG1 for Pax7 AF 647 (1:400): Goat anti-Rabbit IgG for Laminin (1:200) diluted in 3% BSA (in 0.1% PBStw) for 1 h followed by a 30 min incubation with DAPI (5 μg/mL PBS) to visualize nuclei. After washes with PBStw and PBS sections were embedded in Mowiol® and covered with a coverslip. Digital images of stained sections were obtained using an upright microscope with camera (Axio Imager D1, Carl Zeiss, Wrek Göttingen, Germany), controlled by AxioVision AC software (AxioVision AC Rel. 4.8.2, Carl Zeiss Imaging Solutions, Wrek, Wrek Göttingen, Germany) as described previously [9]. Images were quantified using AxioVision 4.8.2 software.

### RNA extraction and qPCR

Total RNA was extracted from each portion of right TA muscle (n = 6/genotype/timepoint) using an RNeasy Fibrous Tissue Mini Kit (Qiagen) as per manufacturer’s instructions. The concentration and quality of RNA samples was determined using Nanodrop 2000 spectrophotometer (Thermo Scientific, Waltham, MA, USA). Real-time RT-PCR was performed as described previously [9], using the forward and reverse primer sequences as described previously [9]. Gene expression was quantified and normalized as described previously [9, 13].

### Antibodies

The following primary antibodies were used throughout the experiments in 5% BSA/TBS/0.1% Tween-20: Rabbit-anti-phosphorylated STAT3 (Y705) (#9131; Cell Signaling Technology, 1:1000), Rabbit-anti-STAT3 (#4904; Cell Signaling Technology, 1:1000), Mouse-anti-myogenin (F5D; #sc12732; Santa Cruz Biotechnology Inc., Dallas, Texas, USA, 1:400), Mouse-anti-Pax7 (developed by A. Kawakami from the Tokyo Institute of Technology and obtained from the Developmental studies hybridoma bank, 1:100), Rabbit-anti-MyoD (M318; #sc760; Santa Cruz, 1:250), and Mouse-anti-MyHC embryonic (F1.652, developed by H. Blau from the Baxter Lab for Stem Cell Biology, Stanford University and obtained from the Developmental studies hybridoma bank, 1:1000). Horseradish peroxidase (HRP)-conjugated donkey-anti-rabbit and sheep-anti-mouse immunoglobulin (GE healthcare life sciences; Marlborough, MA, USA) secondary antibodies were used at 1:5000 in 5% BSA/TBS/0.1% Tween-20.

### Western immunoblotting

For protein analysis the remaining portion of the right TA muscle was homogenized in ice-cold buffer (10 mm Tris-HCl (pH 7.4), 100 mm NaCl, 1 mm EDTA, 1 mm EGTA, 1 mm NaF, 1% Triton, 10% glycerol, 0.1% SDS, 20 mm Na_4_P_2_O_7_, 0.5 mm Na_3_VO_4_, 0.5% sodium deoxycholate, 0.1 mm PMSF and protease and phosphatase inhibitors, all from Sigma-Aldrich). Samples were centrifuged at 10,000 *g* for 5 min at 4°C and the resulting supernatant analyzed for total protein content (DC Protein Assay; Bio-Rad Laboratories, Hercules, CA, USA), with BSA as the standard. Samples were normalized to 2 μg/μl in lysis buffer, resolved in SDS-buffer and heated for 5 min at 95°C. Equal amounts of protein (20 μg per lane) were run on 4–20% Criterion stain-free gels (Biorad) and proteins transferred to 0.45 mm PVDF. Membranes were blocked in 5% BSA in TBS containing 0.1% Tween20 (TBStw) and incubated at 4°C overnight in primary antibody solutions. Horseradish peroxidase (HRP)-conjugated secondary antibodies were applied for 1h at RT. Membranes were visualized with ECL (SuperSignal West Femto Chemiluminescent Substrate, Thermo-Scientific Pierce, IL, USA) and imaged using ChemiDoc MP Imaging System. ECL and total protein stained images were quantified using Image Lab 4.1 software (Bio-Rad Laboratories). To confirm equal loading between lanes, blots were stained and quantified for total protein (Blot-fast Stain; G Biosciences, Maryland Heights, MO, USA) according to manufacturer's instructions. Representative blots are shown in the figures with all western blots and total protein stained membranes used to generate graphs shown in S1 Fig.

### Statistical analyses

All values are presented as mean ± standard error of mean (SEM). Data were analyzed between groups for the effect of genotype and time using a two-way ANOVA with Bonferroni’s post-hoc multiple comparisons test used to detect significant differences between means where appropriate. A *P* value less than 0.05 was considered statistically significant. All statistical analyses were carried out using Prism GraphPad 6 software (GraphPad Software Inc., La Jolla, CA, USA).

## Results

### Deletion of SOCS3 in muscle stem cells disrupts muscle repair after myotoxic injury but does not affect muscle function

**Fig 1:**
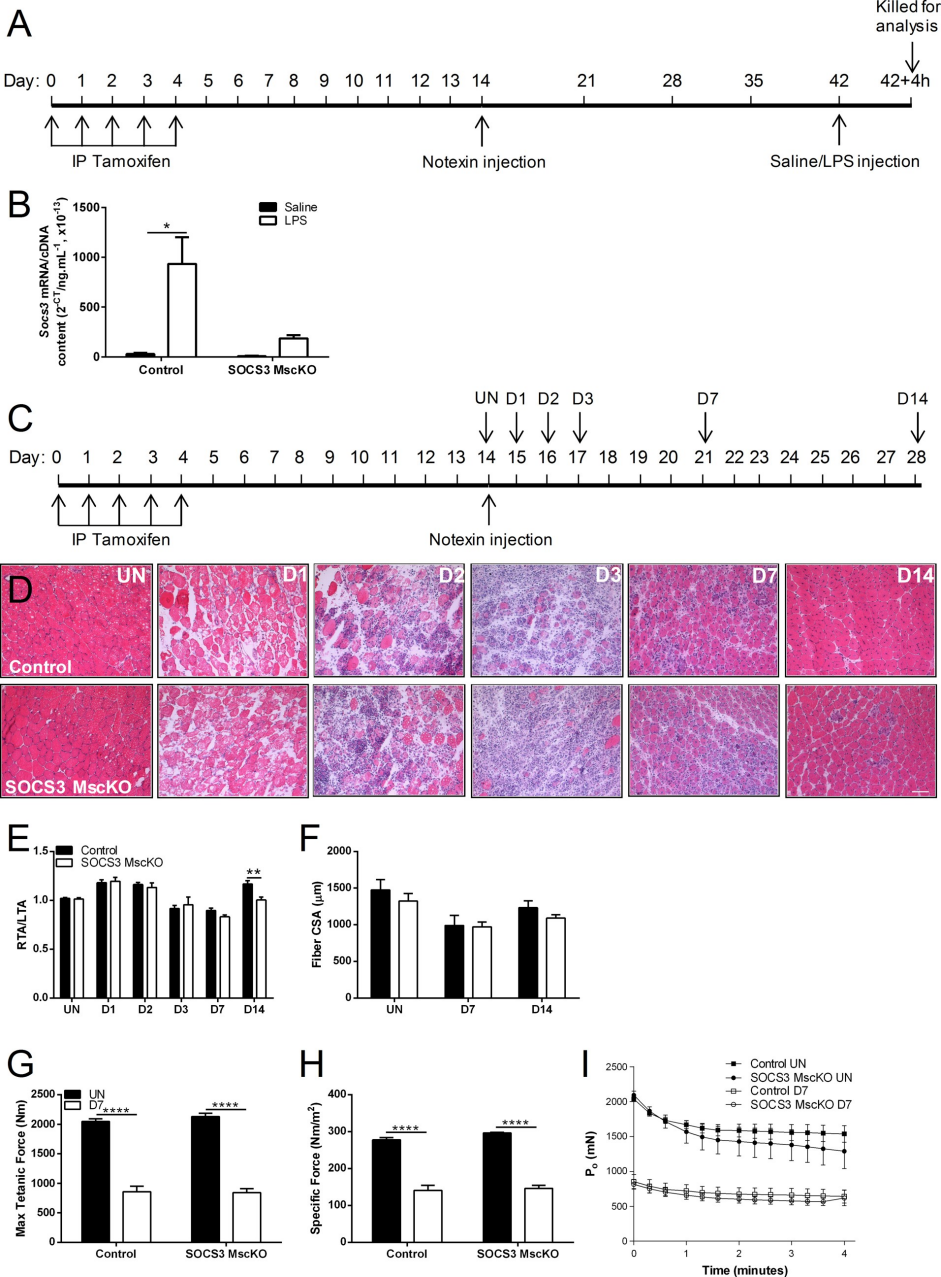
SOCS3 deletion in muscle stem cells *in vivo* delays regeneration after myotoxic injury. (A) Tamoxifen-treated control (SOCS3fl/fl Pax7-CreER-) and SOCS3 MscKO (SOCS3fl/fl Pax7-CreER+) mice received a single 40 μL injection of notexin (10 μg/ml) into the right TA muscle and were allowed to recover for 28 days. On day 28 post-injury, mice received an *i*.*p*. injection of either saline or lipopolysaccharide (LPS; 1 mg/kg). Four hours post-injection, mice were killed and the left and right TA muscles frozen for biochemical analysis. (B) qRT-PCR using primers to detect *Socs3* gene expression in RNA extracted from TA muscles isolated from saline or LPS-injected control and SOCS3 MscKO mice. Data are expressed as mean ± SEM and compared with a two-way ANOVA and Bonferroni’s post-hoc multiple comparisons test to determine the effect of genotype and LPS injection (n = 2 mice/genotype). *P < 0.001. (C) Tamoxifen-treated control (SOCS3^fl/fl^ Pax7-CreER^-^) and SOCS3 MscKO (SOCS3^fl/fl^ Pax7-CreER^+^) mice were either left uninjured (UN) or received a single 40 μL injection of notexin (10 μg/ml) into the right TA muscle and were killed for analysis at 1 day (D1), 2 days (D2), 3 days (D3), 7 days (D7) or 14 days (D14) post-notexin injury. (D) Representative hematoxylin and eosin stained sections of TA muscle from uninjured and injured control and SOCS3 MscKO mice. Scale bar = 100 μm. Muscle mass relative to the uninjured left TA muscle (E) and muscle fiber cross sectional area (CSA; F) were measured at each time-point post-injury. Maximum isometric force (G) and specific (normalized) force (H) were determined at day 7 post-notexin injury. Absolute force production during a 4-minute fatiguing protocol comparing uninjured and day 7 injured right TA muscles from control and SOCS3 MscKO mice (I). Data are expressed as mean ± SEM. Statistical analysis was performed using a two-way ANOVA with a Bonferroni’s post-hoc multiple comparisons test to determine effects of genotype and injury. n = 6 mice/time-point/genotype. **P < 0.01, ****P < 0.0001 compared to uninjured muscles.

SOCS3^fl/fl^ Pax7-CreER mice (SOCS3 MscKO), in which the *Socs3* gene is deleted only in cells expressing Pax7 following administration of tamoxifen, were used to determine whether absence of SOCS3 in muscle stem cells impairs muscle fiber regeneration after myotoxic damage. Skeletal muscle is a heterogenous tissue comprised of multiple cell types that express SOCS3 including mature muscle fibers, immune/inflammatory cells, fibroblasts, and the muscle stem cells, making it difficult to isolate a pure population of SOCS3-deficient cells. We therefore devised a strategy where we ablated the mature muscle fibers in the TA muscles of tamoxifen-treated control and SOCS3 MscKO mice by notexin-injury and allowed the muscle to regenerate for 28 days (Fig 1A), resulting in the formation of either SOCS3 control or SOCS3 deficient muscle fibers from the respective muscle stem cell pools. The efficacy of the tamoxifen-inducible Pax7-CreER mediated SOCS3 deletion was confirmed in these mice by qPCR and identified a lipopolysaccharide (LPS)-induced increase in *Socs3* gene expression in TA muscles at D28 post-notexin injury from control but not SOCS3 MscKO mice (Fig 1B), indicating successful deletion of SOCS3 in the muscle stem cell population of tamoxifen-treated SOCS3^fl/fl^ Pax7-CreER mice.

To examine the role of SOCS3 in the muscle stem cell during muscle repair, tamoxifen-treated control and SOCS3 MscKO mice were either left uninjured (UN) or received a single injection of notexin into the right TA muscle and then killed at D1, D2, D3, D7, or D14 post-injury (Fig 1C). Myofiber degeneration and mononuclear cell infiltration was confirmed at D1, D2 and D3 by hematoxylin and eosin staining (Fig 1D). Injured muscles from SOCS3 MscKO muscles were smaller than injured muscles from control muscles at D14 post-notexin injury (Fig 1E; ** *P* < 0.01). There was no difference in the average muscle fiber cross-sectional area (CSA) between control and SOCS3 MscKO mice at any timepoint (Fig 1F).

We previously reported that muscle fiber-specific deletion of SOCS3, using muscle creatine kinase (MCK)-mediated SOCS3 deletion, increased skeletal muscle fatigue in response to a repeated contraction protocol [9]. Here we examined the force producing capacity of muscles from uninjured and D7 injured control and SOCS3 MscKO mice. Deletion of SOCS3 in the muscle stem cell had no effect on maximal force production (Fig 1G), specific force (Fig 1H) or fatigue response (Fig 1I) in uninjured muscles or in muscles at D7 post-notexin injury.

### Deletion of SOCS3 in muscle stem cells does not alter the muscle inflammatory response after myotoxic injury

**Fig 2:**
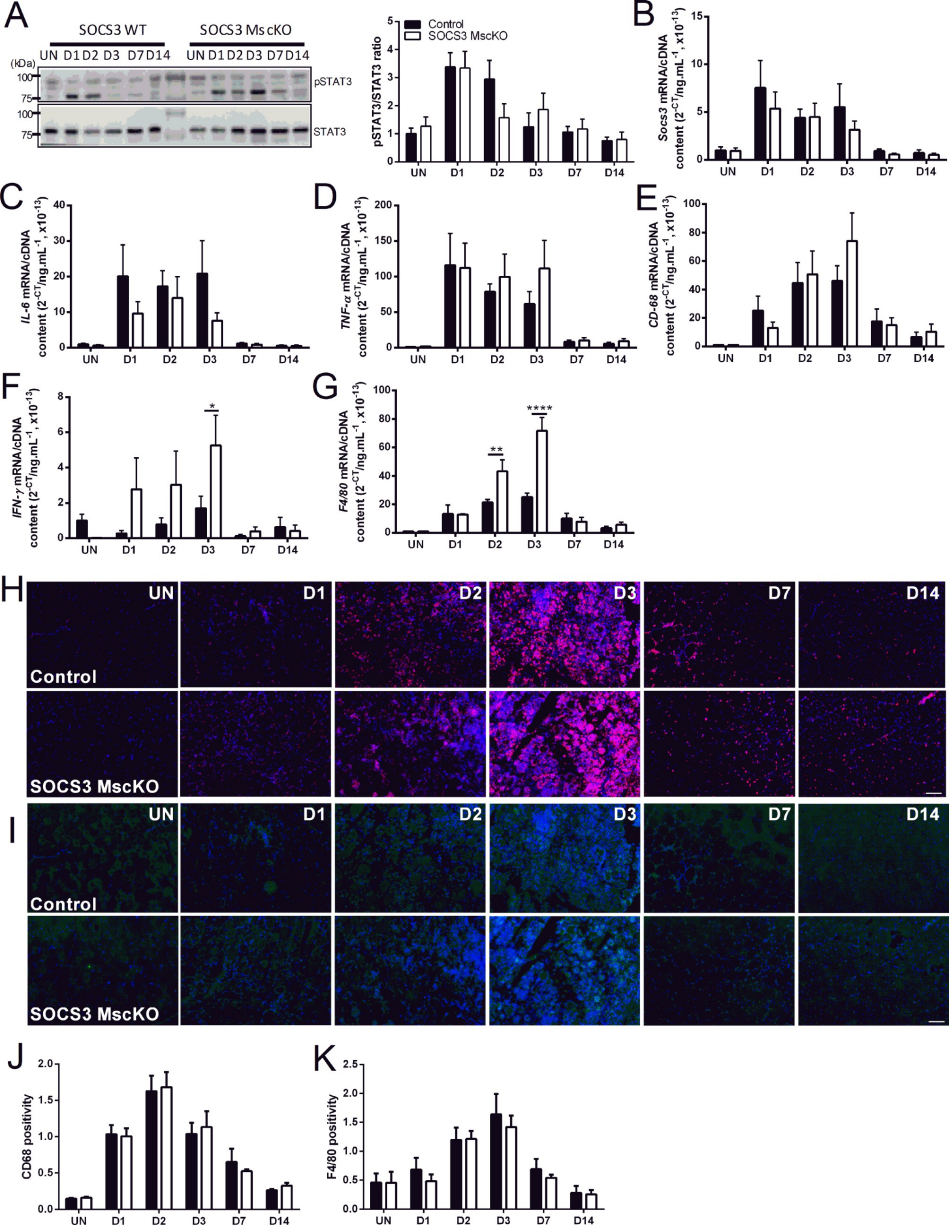
Loss of SOCS3 in muscle stem cells *in vivo* does not affect the inflammatory response after myotoxic injury. Tamoxifen-treated control (SOCS3^fl/fl^ Pax7-CreER^-^) and SOCS3 MKO (SOCS3^fl/fl^ Pax7-CreER^+^) mice were left uninjured (UN) or received a 40 μL injection of notexin (10 μg/ml) into the right TA muscle. Mice were killed for analysis at 1 (D1), 2 (D2), 3 (D3), 7 (D7) or 14 days (D14) post-notexin injury. Protein was extracted from right TA muscles after sectioning and western immunoblotting for phosphorylated and total STAT3 protein. (A) Representative immunoblots for phosphorylated (top) and total (bottom) STAT3 protein levels are shown. Band intensity was quantified using ImageQuant software (Bio-Rad Laboratories) and the ratio of phosphorylated/total STAT3 protein levels was determined. RNA was extracted from snap frozen muscles following dissection and qRT-PCR performed using primers to detect *Socs3* (B) *IL-6* (C), *TNF-α* (D), *CD68* (E), *IFN-γ* (F) and *F4/80* (G). (H) Representative CD68 (red) and DAPI (blue) immunostained sections of TA muscle from uninjured or day 1, 2, 3, 7, or 14 injured control and SOCS3 MscKO mice. (I) Representative F4/80 (green) and DAPI (blue) immunostained sections of TA muscle from uninjured or day 1, 2, 3, 7, or 14 injured control and SOCS3 MscKO mice. The proportion of CD68 (J) and F4/80 (K) positive nuclei were determined using Axiovision software. Data are expressed as mean ± SEM. Statistical analysis was performed using a two-way ANOVA with a Bonferonni’s post-hoc multiple comparisons test to determine the effects of genotype and time. n = 3–6 mice/time-point/genotype. *P < 0.05, **P < 0.01, ****P < 0.0001 compared to control.

In control and SOCS3 MscKO mice, STAT3 phosphorylation was low in uninjured muscles but increased at D1 and D2 post-notexin injury and decreased to basal levels by D3 (Fig 2A; *P* < 0.0001 injury main effect). No difference in STAT3 phosphorylation was observed between control and SOCS3 MscKO mice (Fig 2A). Like our previous observations,[9] *Socs3* gene expression increased at D1 post-notexin injury and remained high to D3, returning to basal levels by D7 (Fig 2B). No difference was observed in *Socs3* gene expression after injury between control and SOCS3 MscKO mice (Fig 2B).

Gene expression of the inflammatory cytokines *IL-6* (Fig 2C) and *TNF-α* (Fig 2D) and the inflammatory cell marker *CD68* (Fig 2E) was low in uninjured muscles, highly expressed at D1, D2, and D3 post-injury, and decreased to basal levels by D7 (Fig 2C–2E; *P* < 0.0001 injury main effect). SOCS3 deletion had no effect on *IL-6* (Fig 2C), *TNF-α* (Fig 2D), or *CD68* (Fig 2E) expression after injury. The gene expression of the pro-inflammatory cytokine *IFN-γ*, as well as the inflammatory cell marker *F4/80*, was low in uninjured muscles from control and SOCS3 MscKO mice, increased progressively until D3, then reduced to basal levels by D7 post-injury (Fig 2F and 2G; *P* < 0.0001 injury main effect). In muscles from SOCS3 MscKO mice, *IFN-γ* gene expression was higher than in muscles from control mice at D3 post-injury (Fig 2F; * *P* < 0.05). Similarly, *F4/80* gene expression was higher in muscles from SOCS3 MscKO mice compared to muscles from control mice at D2 and D3 post-injury (Fig 2G; ** *P* < 0.01, **** *P* < 0.0001 respectively).

Inflammatory cell markers CD68 (Fig 2H) and F4/80 (Fig 2I) were higher at D2 and D3 after injury in muscles from control and SOCS3 MKO mice, but there was no difference in infiltration of inflammatory cells between SOCS3 MKO and control mice (Fig 2J and 2K; *P* < 0.0001 injury main effect). Together, these results imply that absence of SOCS3 in the muscle stem cell does not affect the muscle inflammatory response after injury.

### Deletion of SOCS3 in muscle stem cells delays but does not impair muscle regeneration

**Fig 3:**
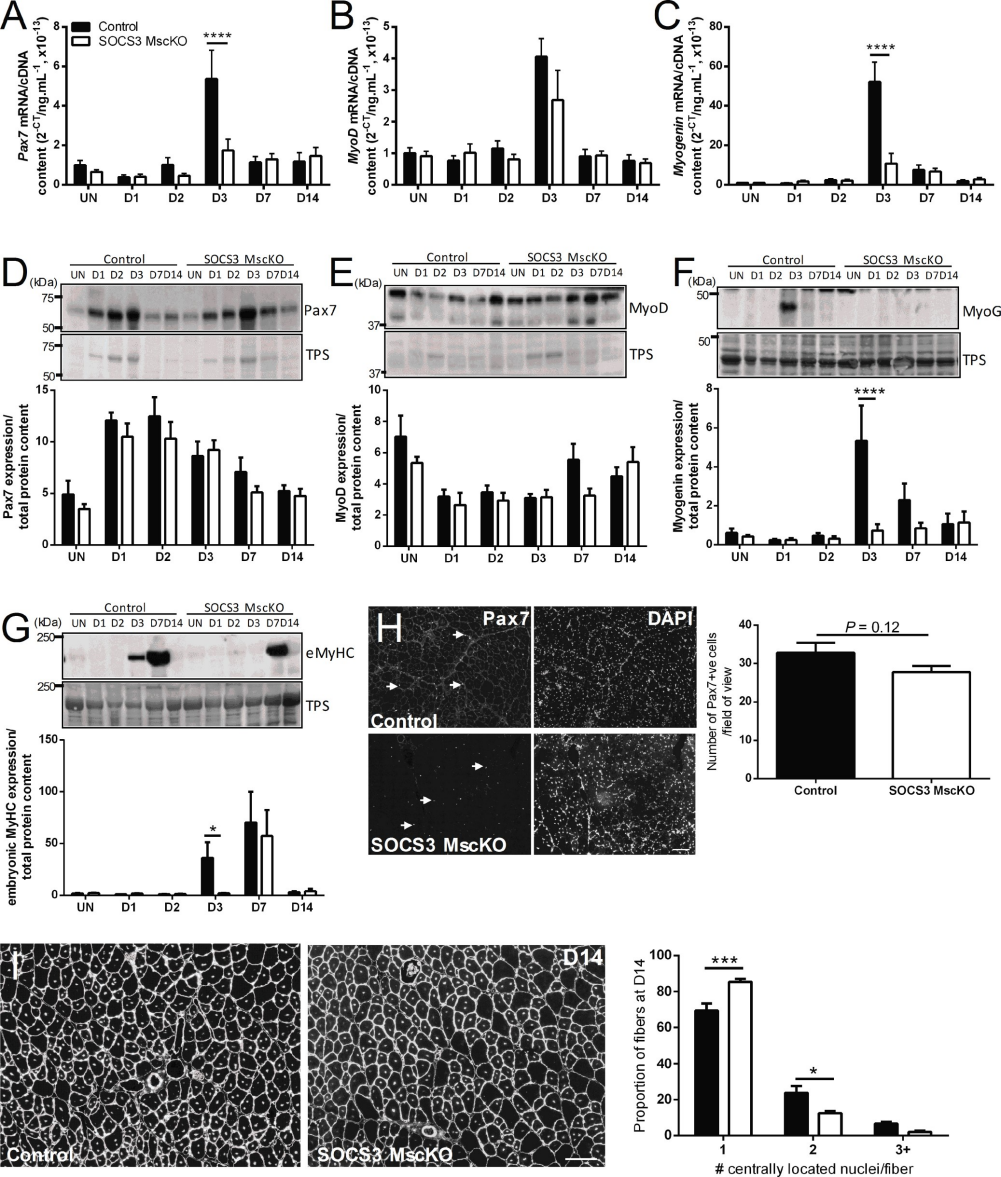
Loss of SOCS3 in muscle stem cells *in vivo* alters the myogenic repair program. Tamoxifen-treated control (SOCS3^fl/fl^ Pax7-CreER^-^) and SOCS3 MKO (SOCS3^fl/fl^ Pax7-CreER^+^) mice were left uninjured (UN) or received a 40 μL injection of notexin (10 μg/ml) into the right TA muscle. Mice were killed for analysis at 1 (D1), 2 (D2), 3 (D3), 7 (D7) or 14 days (D14) post-notexin injury. RNA was extracted from snap frozen muscles following dissection and qRT-PCR performed using primers to detect *Pax7* (A), *MyoD* (B), and *Myogenin* (C). Protein was extracted from remaining OCT embedded right TA muscles after sectioning and western immunoblotting performed. Representative immunoblots for Pax7 (D), MyoD (E), Myogenin (F) and embryonic myosin heavy chain (G) protein levels are shown. Band intensity was quantified using ImageQuant software (Bio-Rad Laboratories) and normalized to total protein levels (TPS). Data are expressed as mean ± SEM. Statistical analysis was performed using a two-way ANOVA with a Bonferroni’s post-hoc multiple comparisons test to determine the effects of genotype and time. n = 5–6 mice/time-point/genotype. *P < 0.05, ****P < 0.0001 compared to control. (H) Representative Pax7 (left) and DAPI (right) immunostained sections of TA muscle from control and SOCS3 MscKO mice at D14 post-injury. The number of Pax7 positive nuclei (as indicated by arrows) was counted per field of view. (I) Representative black and white laminin and DAPI immunostained sections of TA muscle from control and SOCS3 MscKO mice at D14 post-injury. Muscle fibers containing 1, 2, or 3+ -located nuclei were counted per field of view. Data are expressed as mean ± SEM. Statistical analysis was performed using an unpaired Student’s t-test. Scale bar = 100 μm. n = 5–6 mice/time-point/genotype. *P < 0.05, ***P < 0.001 compared to control.

To examine the effect of SOCS3 deletion on muscle regeneration after notexin injury, we analyzed the gene and protein expression of the muscle stem cell marker, *Pax7*, the master myogenic regulator, *MyoD*, the marker of early muscle cell differentiation, *Myogenin*, and the protein expression of embryonic myosin heavy chain (eMyHC). In muscles from control mice, gene expression of *Pax7*, *MyoD*, and *Myogenin* was low in uninjured muscles and at D1 and D2 post-notexin injury, peaked at D3, and subsequently decreased to basal levels by D7 (Fig 3A–3C; *P* < 0.001 injury main effect). Compared to muscles from control mice, *Pax7* gene expression was lower (Fig 3A; ****P < 0.0001), *MyoD* gene expression was not different (Fig 3B), and *Myogenin* gene expression was lower (Fig 3C; ****P < 0.0001) at D3 post-notexin injury in muscles from SOCS3 MscKO mice.

At the protein level, expression of Pax7 (Fig 3D) and MyoD (Fig 3E) was unchanged in muscles from SOCS3 MscKO mice compared to control at all time points after injury, but myogenin expression was lower at D3 (Fig 3F; ****P < 0.0001). Expression of embryonic myosin heavy chain (eMyHC) was blunted at D3 post-notexin injury in muscles from SOCS3 MscKO mice compared to control (Fig 3G; *P < 0.05Immunofluorescence staining showed no change in the number of Pax7+ve muscle stem cells in injured muscles from SOCS3 MscKO mice compared to control (Fig 3H; *P* = 0.12). To determine how altered expression of these myogenic factors affected muscle repair, we next examined the number of centralized nuclei per muscle fiber at D14 post-injury. SOCS3 MscKO muscles contained a higher proportion of fibers with a single central nucleus (Fig 3I; ***P < 0.001) compared to muscles from control mice that contained a higher proportion of fibers with two centralised nuclei (Fig 3I; *P < 0.05). These observations suggest impaired myogenic fusion in muscles of SOCS3 MscKO mice.

**Fig 4:**
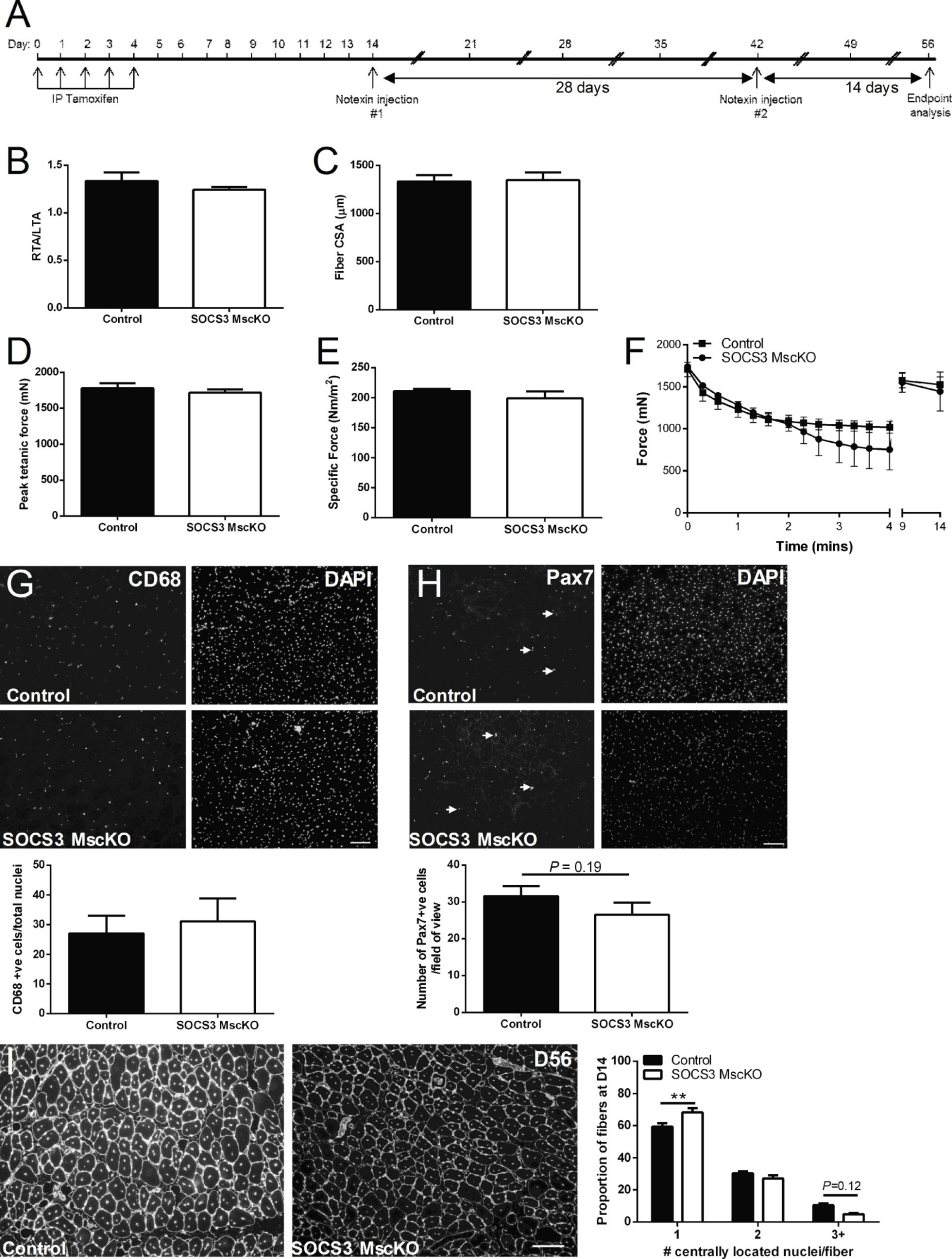
SOCS3 deletion in muscle stem cells does not impair regenerative capacity after sequential myotoxic injuries. Tamoxifen-treated control (SOCS3^fl/fl^ Pax7-CreER^-^) and SOCS3 MscKO (SOCS3^fl/fl^ Pax7-CreER^+^) mice received a single 40 μL injection of notexin (10 μg/ml) into the right TA muscle and were left to recover for 28 days. On day 28 post-injury, all mice received a second single 40 μL injection of notexin (10 μg/ml) into the right TA muscle and were killed for analysis 14 days after the second injury (A). Muscle mass relative to the uninjured left TA muscle (B) and muscle fiber cross-sectional area (CSA; C), maximum isometric force (D), and specific (normalized) force (E) were measured at D14 post the second injury (D56). Data are expressed as mean ± SEM. Statistical analysis was performed using a two-sided Student’s t-test. n = 4–6 mice/time-point/genotype. Force production during a 4-minute fatiguing protocol comparing D56 injured right TA muscles from control and SOCS3 MscKO mice (F). Data are expressed as mean ± SEM. Statistical analysis was performed using a repeated measures two-way ANOVA with a Bonferroni’s post-hoc multiple comparisons test to determine effects of genotype and time. n = 4–6 mice/time-point/genotype. Representative CD68 (left) and DAPI (right) immunostained sections of TA muscle from control and SOCS3 MscKO mice at D56 post-injury. The proportion of CD68 positive nuclei was determined using Axiovision software (G). Representative Pax7 (left) and DAPI (right) immunostained sections of TA muscle from control and SOCS3 MscKO mice at D56 post-injury. The number of Pax7 positive nuclei (as indicated by arrows) was counted per field of view (H). Representative black and white laminin and DAPI immunostained sections of TA muscle from control and SOCS3 MscKO mice at D56 post-injury. Muscle fibers containing 1, 2, or 3+ -located nuclei were counted per field of view (I). Scale bar = 100 μm. Data are expressed as mean ± SEM. Statistical analysis was performed using an unpaired Student’s t-test. n = 6 mice/time-point/genotype. **P < 0.01 compared to control.

We next examined the ability of muscles from control and SOCS3 MscKO mice to regenerate after sequential rounds of myotoxic injury. Tamoxifen-treated control and SOCS3 MscKO mice were either left uninjured (UN) or received a single injection of notexin into the right TA muscle. After 28 days, the mice received a second injection of notexin into the same muscle, and then assessed 14 days later (Fig 4A). The mass of the injured right TA muscle relative to the uninjured left TA muscle was not different (Fig 4B) nor was muscle fiber CSA (Fig 4C) different between muscles of control and SOCS3 MscKO mice. Similar to our findings after a single bout of myotoxic injury, deletion of SOCS3 in the muscle stem cell had no effect on maximal force production (Fig 4D), specific force (Fig 4E) or fatigue (Fig 4F) at D14 after the second bout of notexin injury compared to muscles from control mice. Furthermore, there was no difference in the number of CD68 or Pax7 positive cells in muscles of control and SOCS3 MscKO mice (Fig 4G and 4H). However, consistent with our observations after a single injury, SOCS3 MscKO muscles contained a higher proportion of fibers with a single central nucleus after two sequential injuries (Fig 4I; **P < 0.01) compared to muscles from control mice which showed a trend towards a higher proportion of fibers with more than two centralised nuclei (Fig 4I; *P* = 0.12). Together, these data implicate SOCS3 in myogenic fusion.

## Discussion

It is widely accepted that SOCS3 is expressed by multiple cell types in regenerating skeletal muscles, but its relative contribution to altered muscle inflammation and regeneration has yet to be established. We previously reported that specific deletion of SOCS3 in mature muscle fibers enhanced the inflammatory response after myotoxic injury but did not impair regeneration [9]. Using mice specifically lacking SOCS3 in Pax7-expressing muscle stem cells we have now shown *in vivo* that SOCS3 deletion alters the myogenic program but does not impact overall muscle regeneration post-injury.

A previous study utilized the tamoxifen-inducible Pax7-Cre to selectively ablate Stat3 in the muscle stem cell compartment and demonstrated that conditional ablation of Stat3 increased muscle stem cell expansion during regeneration, but compromised differentiation [3]. This resulted in an increased number of Pax7 positive cells and a reduction in the CSA of the regenerated myofibers [3]. Similar increases in Pax7 positive cells have been observed following administration of Jak/Stat inhibitors in mice [3, 23]. As SOCS3 is a critical negative regulator of STAT3, and SOCS3 deletion results in increased STAT3 activation in all cell types examined to date [16, 17, 24, 25], targeted deletion of SOCS3 in the muscle stem cell would be expected to have the opposite effect to Stat3 deletion; reducing muscle stem cell proliferation and potentially increasing muscle fiber CSA as a consequence of favoured differentiation. Interestingly, our data show that this was not the case and muscle stem cells lacking SOCS3 could regenerate injured muscles despite alterations in the myogenic signaling program.

Analysis of microarray and RNAseq studies performed on freshly isolated (quiescent) versus activated muscle stem cells showed a decrease in *Socs3* gene expression upon activation, suggesting a role for SOCS3 in quiescent muscle stem cells [10, 11]. In contrast, we observed no difference in the number of Pax7 positive cells in muscle sections from control and SOCS3 MscKO mice after a single myotoxic injury, and no change even after two consecutive myotoxic injuries, indicating that SOCS3 is not required to either prevent precocious differentiation or to return muscle stem cells to quiescence. This is supported by analyses incorporating fixation of muscle stem cells prior to isolation that show increased expression is an immediate early response to isolation and that SOCS3 was not higher in truly quiescent muscle stem cells [26].

Despite the deletion of SOCS3 in muscle stem cells, we observed a significant level of *Socs3* gene expression between days 1 and 3 after myotoxic injury that matched the level of expression seen in the muscles from control mice. Interestingly, this pattern of expression mirrored what we have previously reported in mice lacking SOCS3 only in mature muscle fibers [9]. Together these data strongly suggest that this *Socs3* gene expression is likely to come from the infiltrating inflammatory cells present at these timepoints. As inflammation strongly influences the success of muscle regeneration [27], this indicates that SOCS3 may play an important role in inflammatory cells during muscle regeneration, although this has yet to be investigated.

The main finding from this study was that deletion of SOCS3 in the muscle stem cell population delayed expression of the myogenic factors, myogenin and embryonic myosin heavy chain, indicating a role for SOCS3 in myogenic fusion. By assessing the timecourse of myogenic regulatory factor expression we observed a significant blunting in the expression profiles of both myogenin and embryonic myosin heavy chain, suggesting altered myogenic programming. Interestingly, gene expression analyses in myogenin-null muscle stem cells show reduced *Socs3* expression [28], indicating possible cross-regulation between the two genes. Importantly, however, this had little to no impact on the regenerative capacity of muscles from SOCS3 MscKO mice which regenerated successfully after a single injury or consecutive myotoxic injuries.

It is surprising that SOCS3 deletion within the muscle stem cell compartment did not have more of an effect on muscle regeneration given that: 1) Jak/Stat signalling plays a key role in orchestrating the myogenic signalling pathway, particularly promoting myogenic differentiation [3, 5, 29, 30]; and 2) gene knockdown and overexpression in C2C12 cells *in vitro* revealed knockdown of SOCS3 impaired differentiation [5]. However, a potential role for SOCS3 in fusion has been suggested from previous studies using the C2C12 myogenic cell line [5, 8], and Stat3 conditional knockout mice [3]. These previous studies suggested that SOCS3 had a major role in promoting differentiation and fusion, but we show here that genetic deletion of SOCS3 does not impact muscle repair, despite delaying expression of myogenic factors critical for myogenesis. This highlights a functional redundancy between the SOCS protein family members, which explains the lack of phenotype in mice lacking CIS, SOCS5, or SOCS [31]. Although studies in mice with a dual deletion of SOCS1 and SOCS3 suggested no redundancy between the two in the regulation of cytokine signalling in immune cells [32], the interplay between SOCS3 and the other SOCS proteins in muscle cells has yet to be investigated.

Using a tamoxifen-inducible model of genetic deletion we have confirmed *in vivo* that deletion of SOCS3 in the muscle stem cell compartment alters the myogenic program but does not affect overall muscle regeneration after injury. Together with our previous findings in SOCS3 MKO mice [9], we conclude that a reduction in SOCS3 expression in muscle stem cells or muscle fibers does not impair muscle regeneration.

## Supporting information

S1 Fig Full western blot images used to generate representative images and protein expression data. (PDF)

S1 Table All raw data used to generate graphs. (XLSX)
