# Supplementary material for: Deletion of suppressor of cytokine signaling 3 (SOCS3) in muscle stem cells does not alter muscle regeneration in mice after injury
Source: PLoS One. 2019 Feb 27;14(2):e0212880. doi: 10.1371/journal.pone.0212880 (PMC6392323; doi:10.1371/journal.pone.0212880)

S1 Fig. Full western blot images used to generate representative images and protein expression data.

Deletion of Suppressor of Cytokine Signaling 3 (SOCS3) in muscle stem cells alters the myogenic program after injury.

Kristy Swiderski, Marissa K. Caldow, Timur Naim, Jennifer Trieu, Annabel Chee, René Koopman, and Gordon S. Lynch*

Full western blots

Figure 2A

Gel 1

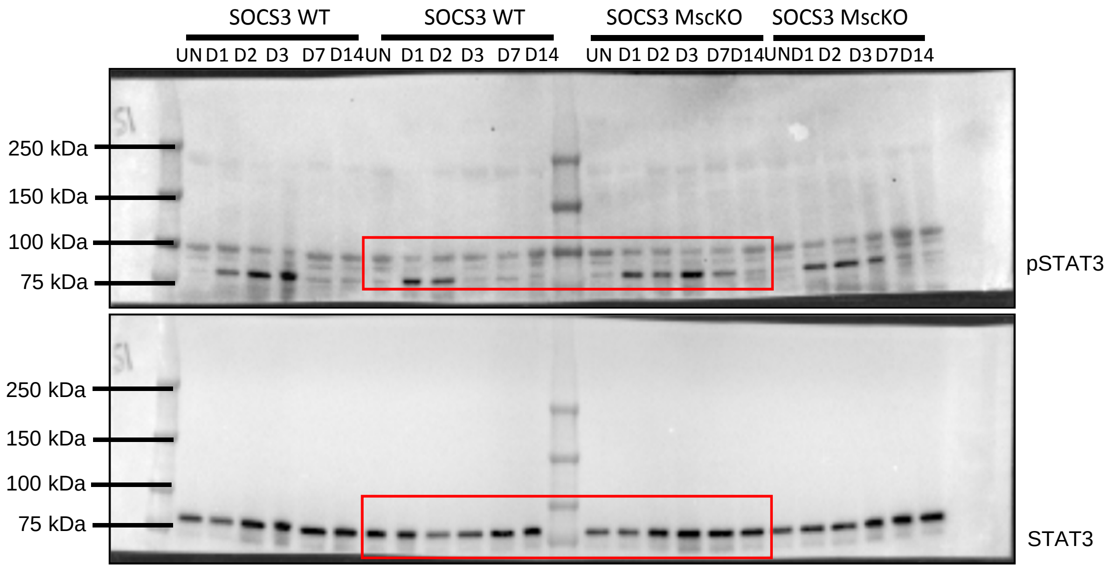

Gel 2

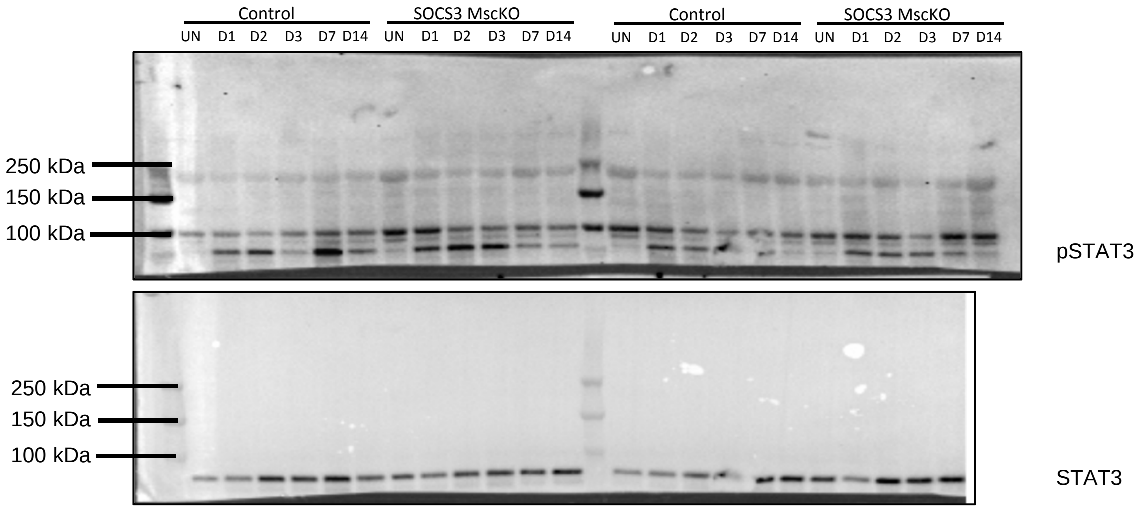

Gel 3

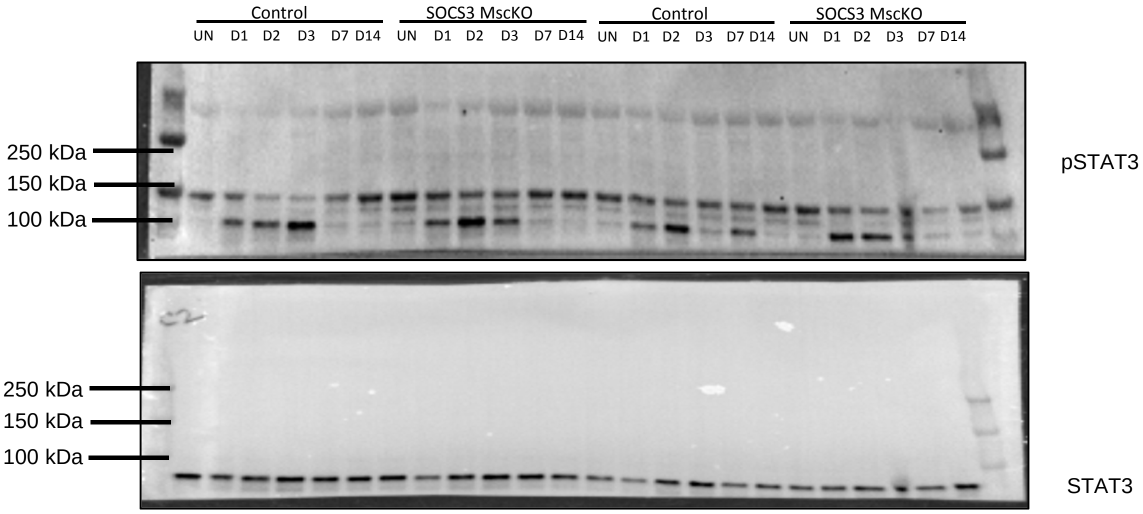

Full western blots
Figure 3D

Gel 1

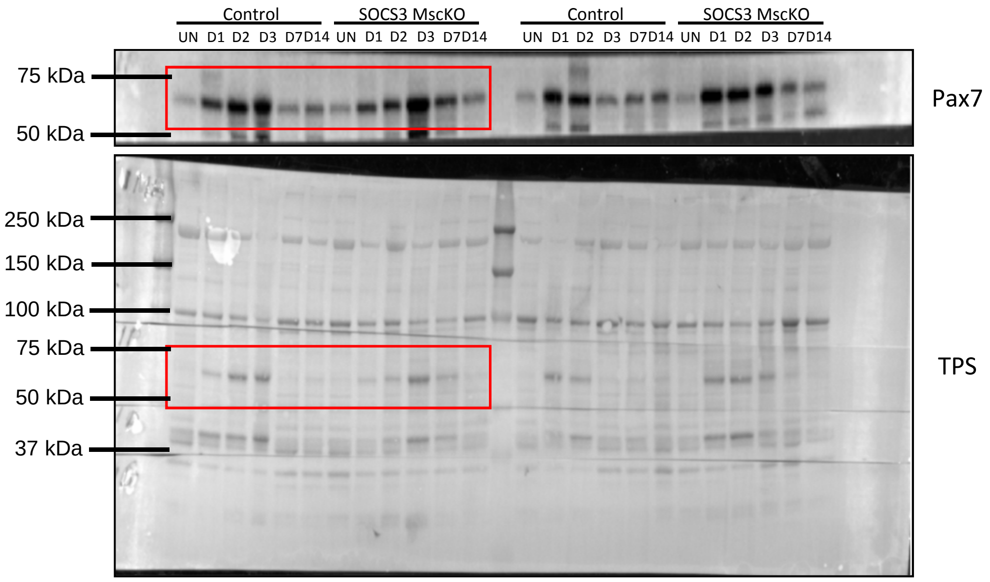

Gel 2

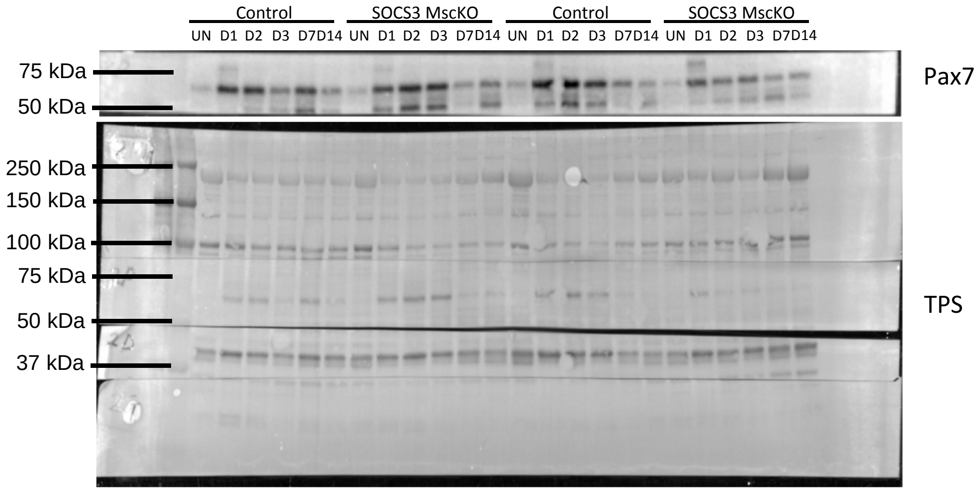

Gel 3

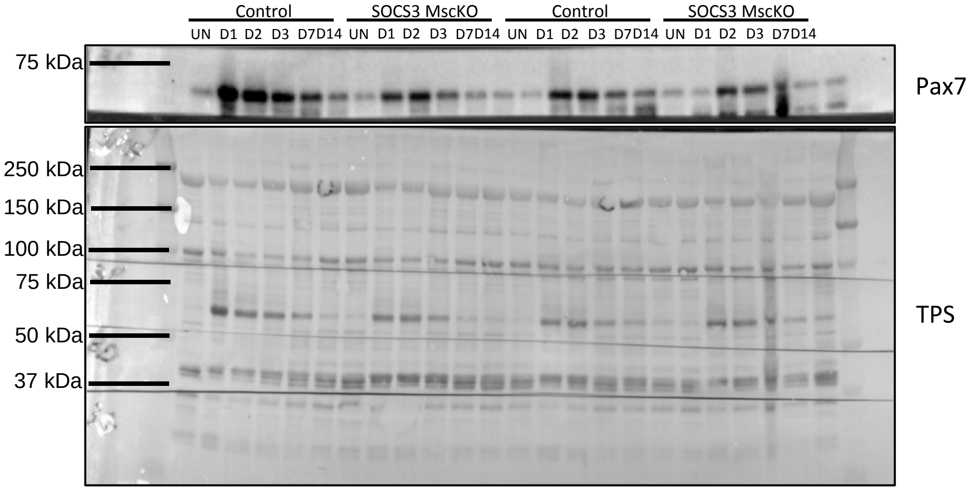

Full western blots
Figure 3E

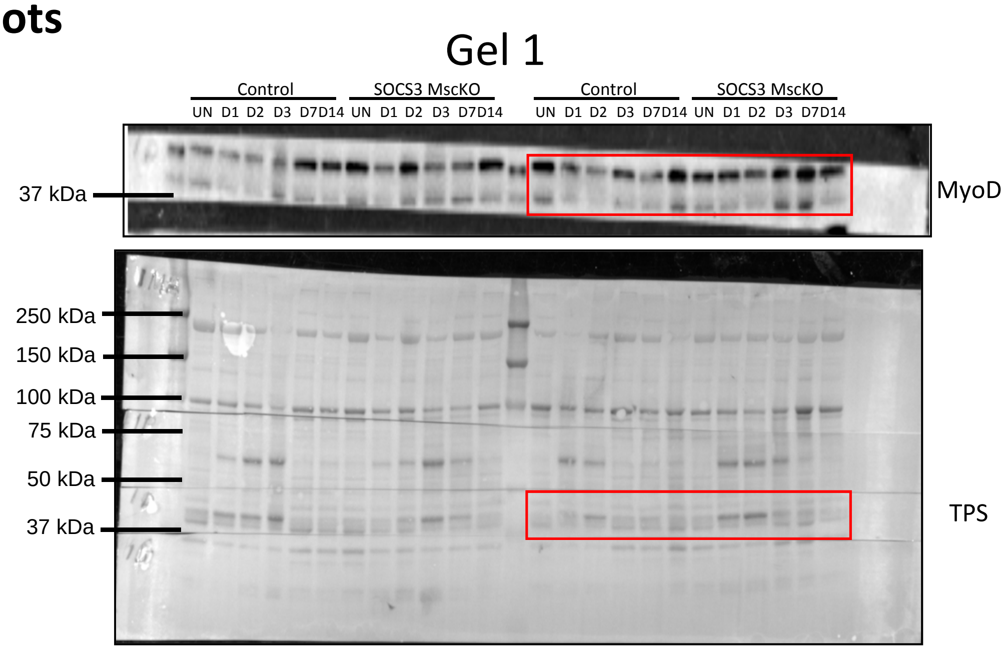

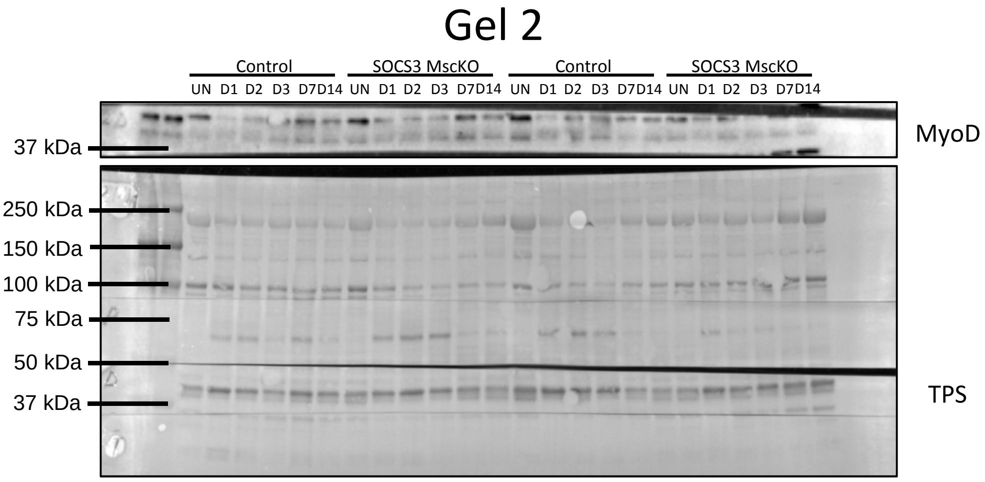

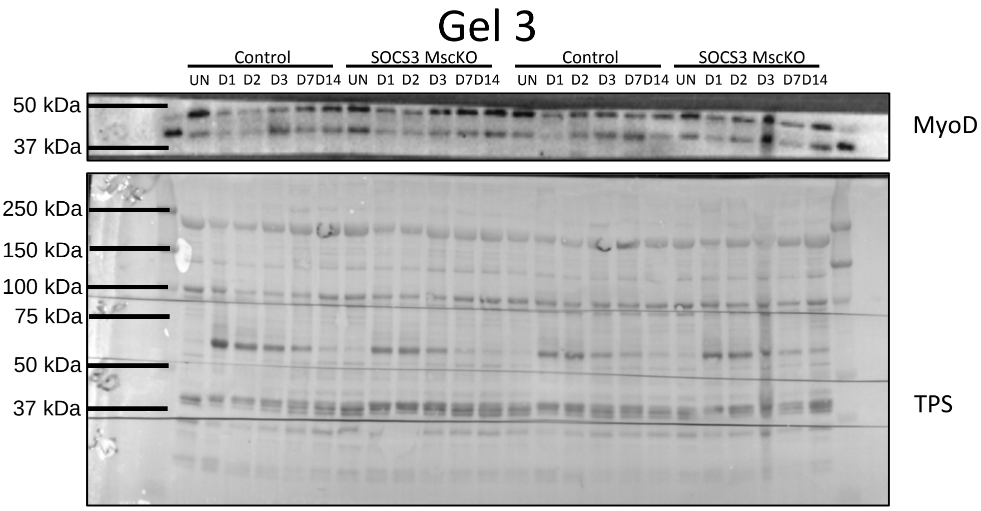

Full western blots
Figure 3F

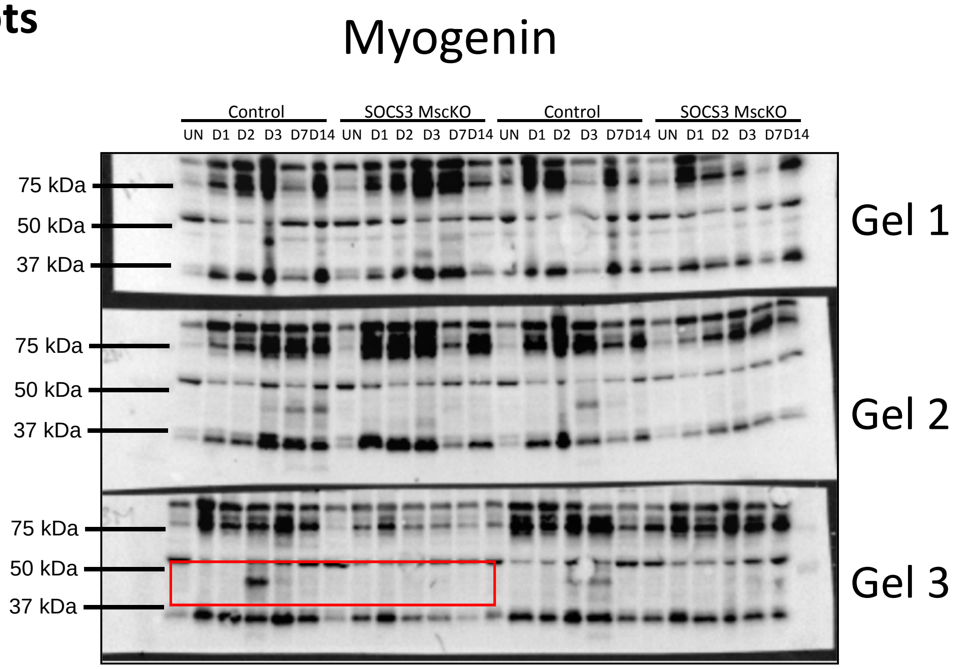

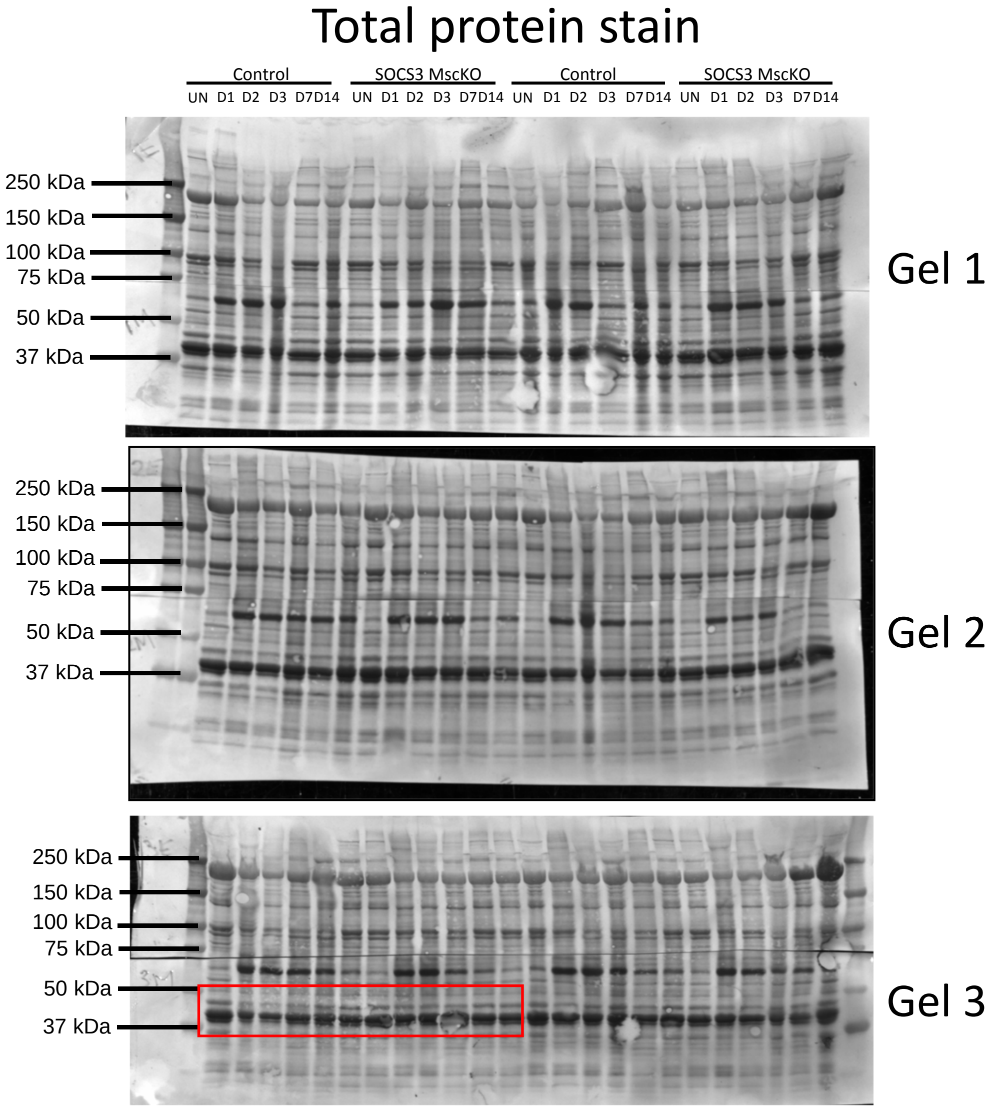

Full western blots
Figure 3G

Embryonic Myosin Heavy Chain

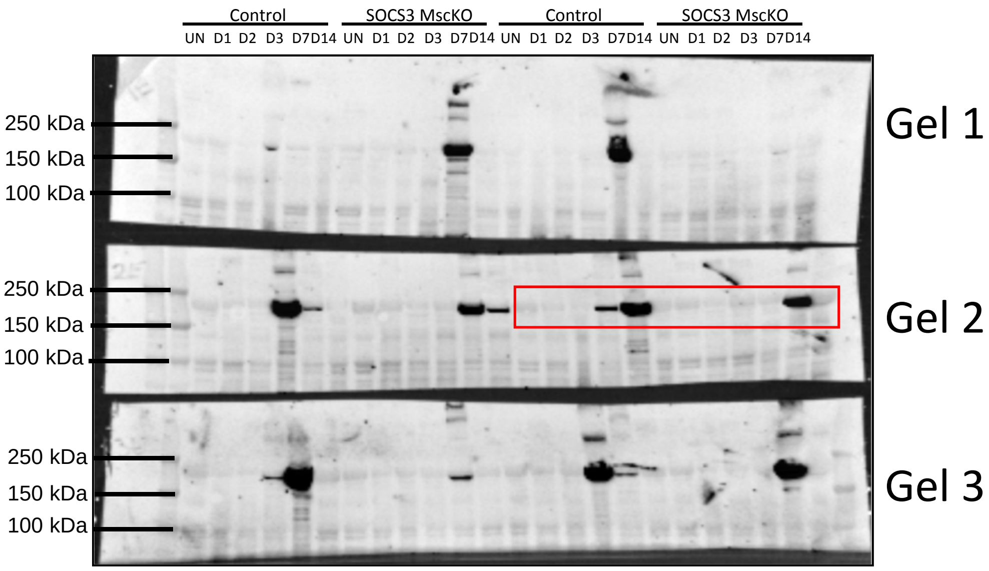

Total protein stain

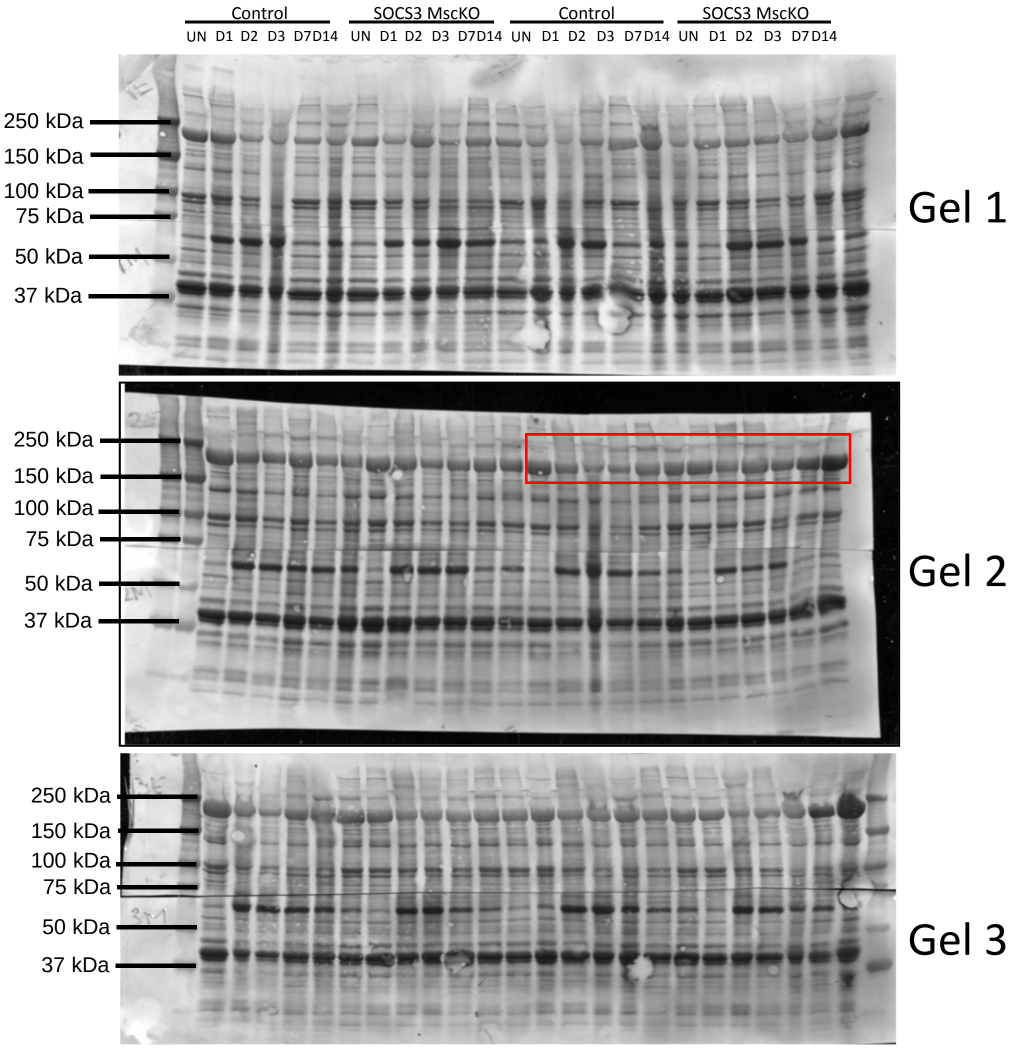

Supplement: S1 Fig — (PDF) [file pone.0212880.s001.pdf]
